# Driving forces of temporal-spatial differences in CO_2_ emissions at the city level for China’s transport sector

**DOI:** 10.1007/s11356-020-12235-4

**Published:** 2021-01-22

**Authors:** Yuxiang Liu, Songyuan Yang, Xianmei Liu, Pibin Guo, Keyong Zhang

**Affiliations:** 1 School of Management and Economics, Beijing University of Technology, Beijing, 100124 China; 2 Norendar International. Ltd., Shijiazhuang, 050021 Hebei Province China; 3 Dept. of Economics, Taiyuan Normal University, Jinzhong, 030619 Shanxi Province China; 4 School of Management and Economics, North University of China, Taiyuan, 030051 Shanxi Province China

**Keywords:** Driving forces, Temporal decomposition, Spatial decomposition, Urban agglomerations, Transport sector

## Abstract

The paper aims to investigate the influencing factors that drive the temporal and spatial differences of CO_2_ emissions for the transportation sector in China. For this purpose, this study adopts a Logistic Mean Division Index (LMDI) model to explore the driving forces of the changes for the transport sector’s CO_2_ emissions from a temporal perspective during 2000–2017 and identifies the key factors of differences in the transport sector’s CO_2_ emissions of China’s 15 cities in four key years (i.e., 2000, 2005, 2010, and 2017) using a multi-regional spatial decomposition model (M-R). Based on the empirical results, it was found that the main forces for affecting CO_2_ emissions of the transport sector are not the same as those from temporal and spatial perspectives. Temporal decomposition results show that the income effect is the dominant factor inducing the increase of CO_2_ emissions in the transport sector, while the transportation intensity effect is the main factor for curbing the CO_2_ emissions. Spatial decomposition results demonstrate that income effect, energy intensity effect, transportation intensity effect, and transportation structure effect are important factors which result in enlarging the differences in city-level CO_2_ emissions. In addition, the less-developed cities and lower energy efficiency cities have greater potential to reduce CO_2_ emissions of the transport sector. Understanding the feature of CO_2_ emissions and the influencing factors of cities is critical for formulating city-level mitigation strategies of the transport sector in China. Overall, it is expected that the level of economic development is the main factor leading to the differences in CO_2_ emissions from a spatial-temporal perspective*.*

## Introduction

Climate change has been regarded as the most serious challenge and core issue faced by humans to achieve sustainable development of the socioeconomic system (Zhu et al. 2019). Climate change mitigation and adaptation need joint endeavors from temporal and spatial perspectives (Tian et al. 2019). Since 2007, China, the world’s largest developing country, has been the largest CO_2_ emitter in the world (Jing et al. 2018), and in 2015, its CO_2_ emissions from energy consumption reached 9265.1 million tons (Mt). China has abided by reducing carbon intensity (i.e., carbon emissions per unit of gross domestic product) reduction of 60–65% below the 2005 level by 2030 at the 2015 Paris Climate Change Conference (UNFCCC 2015). Besides, in order to achieve this object, the State Council of China set up “a blue-sky defense plan” for improving air quality at the city level, which has been considered the Beijing-Tianjin-Hebei region and surrounding areas (two + twenty-six cities^1^), Yangtze River Delta and Fen nutrient-laden plain as the key areas for ensuring success.

The transport sector is one of the major elements of globalization and makes an important contribution to the economy, plays an indispensable role in daily life and work in the whole world (Lin and Xie 2014; Yin et al. 2015). However, large-scale transportation services will consume a great deal of energy, accounting for approximately one-third of the total energy consumption of the world and inevitably produce 23% of CO_2_ emissions (IEA 2017). At present, the transport sector has been identified as one of the major contributors to CO_2_ emissions and degradation of the environment. Besides, it has been regarded as the second-largest energy-consuming sector after the industrial sector (Hao et al. 2014; Yin et al. 2015). In 2015, the global transport sector emitted 2715 Mt of CO_2_, of which China’s transport sector is estimated to account for about one-third. CO_2_ emissions of China’s transport sector reached 781.29 Mt in 2017 which accounted for 7.81% of China’s total CO_2_ emissions (Zhang et al. 2019). Some scholars predicted that China’s transport sector’s energy consumption and CO_2_ emissions will be increased by approximately 50% by 2030 and by 80% by 2050 (Guo et al. 2014; Xu and Lin 2016; Lv et al. 2019). Consequently, it is critical for the policymakers to reduce the total emissions from the transport sector.

Cities are regarded as the main consumers of energy and emitters in the whole world (Shan et al. 2017). According to the International Energy Agency (IEA) (2009), the CO_2_ emissions generated from energy consumption in cities will rise 1.8% per year from 2006 to 2030, with the proportion of global CO_2_ emissions increasing from 71 to 76%. As a result of economic development and improvement of income level and quality of life, the urban population has increased fleetly during recent years. The urban population rose to 750 million by 2014, an approximately 2.5 time increased from 1990. At present, more than half of the population lives in cities (Mi et al. 2016; NBSC 2015). China’s urbanization rate has increased from 17.92% in 1978 to 47.85% in 2015 (Huang et al. 2019). Chinese cities have made a contribution to about 85% of the total CO_2_ emissions, which has been 5% higher than the USA and 16% higher than Europe, respectively (Dhakal 2009; Shan et al. 2017). Besides, the urbanization process will continue at express speed in the next decade (Shan et al. 2017; Li et al. 2019). Therefore, Chinese cities are regarded as the vital role player in considering the CO_2_ emission responsibilities (Shan et al. 2019a). However, most of the previous literature has focused on transport sector CO_2_ emissions at the national level and more provinces even regions of the countries; there is little literature on the city level.

In this study, we investigated the CO_2_ emissions of China’s transport sector at the city level during 2000–2017 from both temporal change and spatial discrepancy’s perspectives. At the first step, we simply described the changing trend of CO_2_ emissions and the characteristics of temporal and spatial of China’s transport sector; at the second step, we deeply explored the influencing factors of CO_2_ emissions at the city level in China’s transport sector based on Logistic Mean Division Index (LMDI) decomposition analysis method from the temporal perspective, and simultaneously compare the differences in CO_2_ emissions of different transport sectors and the impacting factors between the urban agglomeration and national average using M-R spatial decomposition analysis method from the spatial angles; and at the last step, we provided some useful references or policy suggestions for China’s transport sector from the city-level to reduce CO_2_ emissions. Compared with existing studies, this study makes the following contributions: (1) This study applied the improved M-R spatial decomposition analysis model to explore the influencing factors of spatial differences of China’s transport sector; in addition, the decomposition indicator of transportation intensity is introduced into M-R spatial decomposition analysis model for the first time. Thus, this study extended a useful reference method for the problem of spatial difference and developed the decomposition indicators for the transport sector. (2) We analyzed the differences and changes of CO_2_ emissions from the city level and provided a new entry point for the transport sector to control CO_2_ emissions or formulate more accurate emission reduction measures.

## Literature review

The transport sector is a vital pillar of economic and society development. Although many studies have been conducted to explore the CO_2_ emissions from the transport sector in the global (Timilsina and Shrestha 2009; Saboori et al. 2014; Yin et al. 2015), national (Wang et al. 2007; Wang et al. 2011; Zhou et al. 2013; Hao et al. 2014; Tiwari et al. 2020; Liu and Feng 2020), provincial (Xu and Lin 2018; Feng and Wang 2018; Zhang et al. 2019), and region levels (Guo et al. 2014), the transport sector’s CO_2_ emissions of Chinese cities have not been well documented when compared with the mentioned above literature. What’s more, existing studies have been paid more attention to spatial-temporal analysis of the differences in regional CO_2_ emissions in recent years (Huang and Meng 2013; Ang et al. 2016). However, many literatures on spatial analysis mainly focus on the production sector (Yang et al. 2019), household sector (Li et al. 2017), building sector (Chen and Chen 2019), and power sector (Wang et al. 2019), and less literature are involved in the transport sector.

The decomposition method is very popular and widely applied in the field of energy and emissions (Su and Ang 2016; Li et al. 2017; Li et al. 2017), which mainly contain two types: the one is called to structure decomposition analysis (SDA) and the other is called to index decomposition analysis (IDA). The former calculation process is relatively tedious, advocated by Chang and Lin (1998) to explore the key factors of industrial CO_2_ emission changes in Taiwan, and decomposition index changes based on the input-output tables of specific years depending on the input-output model aimed for quantitative economic assessment. The latter method was first employed by Hulten (1973) to analyze energy consumption which is applied to explore the forces of changes in CO_2_ emissions and to provide relevant suggestions for carbon mitigation. In addition, the second is better than the first method in the accessibility of data; thus, the IDA approach is more widely employed than the SDA (Liu et al. 2007; Zhu et al. 2017).

LMDI, proposed by Ang and Choi (1997), is the most mature model used in IDA among them, which is widely used in many fields based on its greater advantages; in addition, it has more applicability and more interpretation of results than other decomposition models (Xu et al. 2014; Zhu et al. 2017). Wang et al. (2011) investigated the potential factors influencing the changes of the transport sector’s CO_2_ emissions based on the LMDI model and found that the effect of per capita economic activity is primarily responsible for driving transport sector CO_2_ emission growth, while the transportation intensity effect is the main factor of CO_2_ emission reduction. Timilsina and Shrestha (2009) explored the driving factors of growth in CO_2_ emissions in the transport sector. They found that economic growth, population growth, and energy intensity were the main reasons of CO_2_ emissions; thus, they suggested that the local government should adopt fiscal measures to encourage the use of new energy fuels. In a later study, Guo et al. (2014) revealed the CO_2_ emission features for the transport sector in 30 Chines provinces and then quantified the related driving forces by using the time-series LMDI method. They found that CO_2_ emissions were mainly contributed by both economic activity effect and population effect, while energy structure had a marginal effect. And the latter literature has investigated the determinants of CO_2_ emissions caused by the transport sector from 12 European countries and Pakistan, and they found that the difference in CO_2_ emissions is largely the same (Raza and Lin 2020; Georgatzi et al. 2020).

A multi-regional (M-R) spatial decomposition model, proposed by Ang 2015can be used to describe the reasons that cause the differences in CO_2_ emissions among countries or among various regions within the same country. This model can reduce decomposition cases, avoiding subjectivity in basic region option, maintaining proper circularity, and supporting valuable information considering the potential of energy-saving and emission-reduction (Li et al. 2017). Therefore, this paper employs the M-R spatial decomposition model to explore the differences in CO_2_ emissions of the transport sector.

The rest of this study is organized as follows. The “Literature review” section described the literature review and the “Methodology, study area, and data” section proposes the methodology, including LMDI temporal decomposition model and multi-regional M-R spatial decomposition model; meanwhile, data collection sources were also involved. The results of LMDI decomposition and M-R decomposition for the urban agglomerations in 2000–2017 are illustrated and the underlying causes and policy implications are discussed in the “Results and discussion” section. The “Conclusions” section concludes the article.

## Methodology, study area, and data

### Study areas

**Fig. 1:**
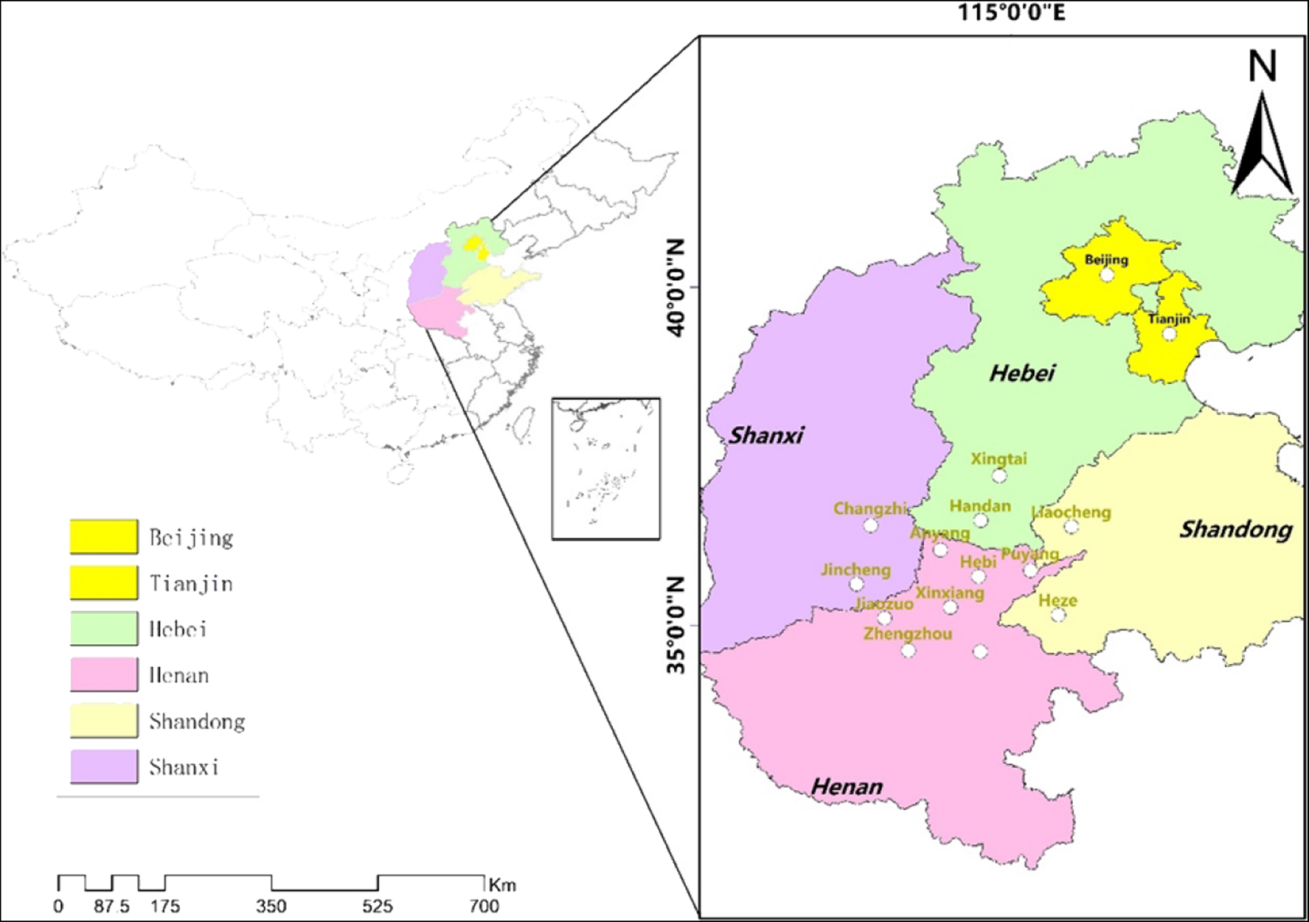
The 15 east-central Chinese cities within Beijing-Tianjin-Hebei region and surrounding areas of urban agglomerations (namely Beijing, Tianjin, Handan, Xingtai, Liaocheng, Heze, Zhengzhou, Puyang, Kaifeng, Hebi, Xinxiang, Jiaozuo, Jincheng, Changzhi, and Anyang)

In this study, we selected 15 cities from four east-central provinces and two municipalities (Fig. 1) (including Beijing, Tianjin, Hebei, Henan, Shandong, and Shanxi). These 15 cities are affiliated with the Beijing-Tianjin-Hebei region and surrounding areas, which are considered one of the key areas in a blue-sky defense plan. As urban units, their growth rates do not differ much, and the data are not very different, but as the major cities in the blue-sky program, these cities approximately account for 26.32% of the nation’s total GDP and 29.23% of its total carbon emissions; in other words, only when we control the transport sector’s CO_2_ emissions of these cities can we win the battle for the blue-sky plan.

The reasons as follows: (1) the absolute CO_2_ emissions of these cities in the transport sector have not been got much attention for alleviating CO_2_ emissions. Furthermore, Chinese cities have encountered constraints in data availability (Guan et al. 2017; Tian et al. 2019) and quality (Tian et al. 2019). (2) A few pieces of literature explore the CO_2_ emissions transport sector at the city level from temporal and spatial perspectives. (3) The 15 cities as part of the blue-sky defense plan have research value; besides, the total share of the population for these aggregated 15 cities compared to the population of national has increased from 13.29% in 2000 to 15.29% in 2017, the percentage of GDP increased from 6.47% in 2000 to 9.71% in 2017, and the percentage of CO_2_ emissions of the transport sector has increased from 6.32% in 2000 to 8.10% in 2017. The basic information about these cities is presented in Table 1.

**Table 1:** The socio-economic characteristics of 15 east-central Chinese cities in 2017

Cities	GDP (10^8^ Yuan)	Area (km^2^)	Population (10^4^ persons)	GDP per capita (Yuan/capita)	Population density (persons/km^2^)
Anyang	2393.22	7352	624	46,450	839.23
Hebi	861.90	2182	170	53,063	774.52
Jiaozuo	2371.50	4071	371	66,328	913.78
Kaifeng	2002.23	6444	559	43,936	859.71
Puyang	1654.48	4188	432	45,644	1024.36
Zhengzhou	10,143.37	7446	842	101,349	1087.83
Xinxiang	2526.26	8666	647	43,700	735.06
Handan	3454.58	12,065	1051	36,289	870.29
Xingtai	2150.76	12,433	790	29,210	627.36
Liaocheng	3152.52	8984	640	51,935	692.34
Heze	3078.79	12,256	1019	35,184	818.37
Changzhi	1645.15	13,896	338	47,540	242.52
Jincheng	1351.86	9425	221	57,819	232.36
Beijing	30,319.90	16,411	1359	140,211	819.57
Tianjin	18,809.94	11,917	1050	120,711	861.79

### Decomposition model

#### Estimation of CO_2_ emissions

This study mainly analyzes four modes of transportation in each city: highway, railway, waterway, and civil aviation; the specific calculation method is described following Zhao et al. (2016). Based on Eq. (16), the amount of energy consumption is measured by 10^4^ tons of standard coal equivalent (10^4^ tce).

1 $$ {C}^t=\sum \limits_{i=1}^4{C}_i^t=\sum \limits_{i=1}^4\sum \limits_{j=1}^9{A}_{ij}^t\times {R}_{ij}^t\times {F}_j $$

where *C* ^t^ denotes the total CO_2_ emissions of the year *t* in the transportation sector; *R*_*ij*_ denotes energy consumption per unit service of the *i*th transportation mode based on *j*th energy in year *t*; and *F*_*j*_ denotes the emissions coefficient of the *j*th energy resource.

#### Kaya identity

Kaya identity, a systematic and integrated method, is regarded as a popular tool to uncover demographic, economic, energetic, and environmental associations (Wang and Li 2019). In this study, the extended Kaya identity is adopted to analyze the driving forces of CO_2_ emissions of the transport sector. City-level CO_2_ emissions in China can be decomposed into six kinds of driving factors, as shown in Eq. (1).

2 $$ {\displaystyle \begin{array}{c}{C}_i=\sum \limits_{j=1}^4\sum \limits_{k=1}^9{C}_{ij k}\\ {}\kern1em =\sum \limits_{j=1}^4\sum \limits_{k=1}^9\frac{C_{ij k}}{E_{ij k}}.\frac{E_{ij k}}{A_{ij}}.\frac{A_{ij}}{A_i}.\frac{A_i}{G_i}.\frac{G_i}{P_i}.{P}_i\\ {}\kern1em =\sum \limits_{j=1}^4\sum \limits_{k=1}^9E{S}_{ij k}.E{I}_{ij}.A{S}_i.A{I}_i.A{G}_i.T{P}_i\end{array}} $$

where *i* denotes each city in China; *j* denotes the sector involved in this study (*j* = 1,2,3,4 for road, railway, waterway, and civil aviation); and *k* denotes the energy type consumed by each sector (*k* = 1, 2…,9 such as raw coal, coke, crude oil, gasoline, kerosene, diesel oil, fuel oil, natural gas, and electricity).

The variables considering the temporal and spatial decomposition models are defined in Tables 2 and 3.

**Table 2:** Symbol of variables involved in this study

Variable	Definition	Unit	Variable	Definition	Unit
*C*_*i*_	The total amount of CO_2_ emissions of sector *i*	10^4^ tons	*ES*_*ijk*_	*F*_*ijk*_ = *C*_*ijk*_/*E*_*ijk*_, carbon emission coefficient for *k*th fossil energy of sector *j* in city *i*	t CO_2_/tce
*C*_*ijk*_	CO_2_ emissions of *k*th fossil energy consumption by sector *j* in city *i*	10^4^ tons	*EI*_*ij*_	*EI*_*ij*_ = *E*_*ij*_/*A*_*ij*_, the energy intensity of sector *j* in city *i*	Kg ce/10^4^ t-kilometer
*E*_*ijk*_	*k*th fossil energy consumption of sector *j* in city *i*	10^4^ tce	*AS*_*i*_	*AS*_i_ = *A*_*ij*_/*A*_*i*_, the output share of sector *j* in city *i*	%
*A*_*ij*_	Transportation service of sector *j* in city *i*	10^4^t-kilometer	*AI*_*i*_	*AI*_*i*_ = *A*_*i*_/*G*_*i*_, transportation intensity of city *i*	Ton-kilometer/10^4^ yuan
*A*_*i*_	Transportation service in city *i*	10^4^t-kilometer	*AG*_*i*_	*AG*_*i*_*=G*_*i*_ / *A*_*i*_, GDP per capita in city *i*	10^4^ yuan
*G*_*i*_	The gross domestic product in city *i*	10^4^ yuan	*C*	The total amount of CO_2_ emissions	10^4^ ton
*P*_*i*_	The total population in city *i*	10^4^ persons

**Table 3:** Definition of various variables in Eq. (9)

Variables	Definition
Δ*C*_*ES*_	*Energy structure effect*, representing the change of CO_2_ emissions due to the change of energy structure
Δ*C*_*EI*_	*Energy intensity effect*, representing the change of CO_2_ emissions due to the change of energy efficiency of the transport sector
Δ*C*_*AS*_	*Transportation structure effect*, representing the change of CO_2_ emissions due to the change of output share of the transport sector
Δ*C*_*AI*_	*Transportation intensity effect*, representing the change of CO_2_ emissions due to the change of transportation efficiency
Δ*C*_*AG*_	*Income effect*, representing the change of CO_2_ emissions due to the improvement of people’s income level
Δ*C*_*TP*_	*Population scale effect*, representing the change of CO_2_ emissions due to the population growth

*Note*: The difference between M-R model and LMDI model that mainly exists in LMDI was applied in exploring the changes of CO_2_ emissions for the degree of time and the M-R model was applied in analyzing the difference among cities. However, there is a similar theoretical basis in them

#### Temporal-LMDI framework

The aggregate changes of CO_2_ emissions for each city of China between the base year 0 and the target year *T* are decomposed into the driving factors of energy structure, energy intensity, transportation structure, transportation intensity, income, and population scale using the additive technique proposed by Ang (2005). As shown in Eq. (2),

3 $$ {\displaystyle \begin{array}{c}\varDelta {C}_{i, tot}^{T-0}={C}_i^T-{C}_i^0\\ {}\kern2.34em =\varDelta {C}_{i, ES}^{T-0}+\varDelta {C}_{i, EI}^{T-0}+\varDelta {C}_{i, AS}^{T-0}+\varDelta {C}_{i, AI}^{T-0}+\varDelta {C}_{i, AG}^{T-0}+\varDelta {C}_{i, TP}^{T-0}\end{array}} $$

The effects of differences driving factors of city CO_2_ emissions of the transport sector are calculated by the following equations:

4 $$ \varDelta {C}_{i, ES}^{T-0}=\sum \limits_{j=1}^4\sum \limits_{k=1}^9L\left({C}_{ijk}^T,{C}_{ijk}^0\right).\ln \left(\frac{F_{ijk}^T}{F_{ijk}^0}\right) $$

5 $$ \varDelta {C}_{i, EI}^{T-0}=\sum \limits_{j=1}^4\sum \limits_{k=1}^9L\left({C}_{ij k}^T,{C}_{ij k}^0\right).\ln \left(\frac{E{I}_{ij}^T}{E{I}_{ij}^0}\right) $$

6 $$ \varDelta {C}_{i, AS}^{T-0}=\sum \limits_{j=1}^4\sum \limits_{k=1}^9L\left({C}_{ijk}^T,{C}_{ijk}^0\right).\ln \left(\frac{A{S}_i^T}{A{S}_i^0}\right) $$

7 $$ \varDelta {C}_{i, AI}^{T-0}=\sum \limits_{j=1}^4\sum \limits_{k=1}^9L\left({C}_{ijk}^T,{C}_{ijk}^0\right).\ln \left(\frac{A{I}_i^T}{A{I}_i^0}\right) $$

8 $$ \varDelta {C}_{i, AG}^{T-0}=\sum \limits_{j=1}^4\sum \limits_{k=1}^9L\left({C}_{ijk}^T,{C}_{ijk}^0\right).\ln \left(\frac{A{G}_i^T}{A{G}_i^0}\right) $$

9 $$ \varDelta {C}_{i, TP}^{T-0}=\sum \limits_{j=1}^4\sum \limits_{k=1}^9L\left({C}_{ijk}^T,{C}_{ijk}^0\right).\ln \left(\frac{T{P}_i^T}{T{P}_i^0}\right) $$

where $L\left({C}_{ijk}^T,{C}_{ijk}^0\right)=\frac{C_{ijk}^T-{C}_{ijk}^0}{\ln {C}_{ijk}^T-\ln {C}_{ijk}^0}$ is the logarithmic mean weight.

#### Spatial-M-R framework

Spatial decomposition analysis has been paid attention to multi-county comparisons of energy consumption or CO_2_ emissions using IDA. Such a way often sees large variations in the data for the factors in the IDA identity. It is different from the conventional temporal decomposition analysis using time-series data or the data of two different years of a country.

The spatial decomposition model shows advantages in comparing the differences in many fields, i.e., energy consumption, energy efficiency, or CO_2_ emissions among regions within a country (Li et al. 2017). In this study, we chose each target city to compare with a benchmark reference entirety (national average) considering the arithmetic average of the national group. The differences of CO_2_ emissions of the transport sector between cities *i* and national average *R*_*u*_, denoted as $\Delta {C}_{t, tot}^{\left({R}_i-{R}_u\right)}$, expressed as Eq. (9):

10 $$ {\displaystyle \begin{array}{c}\varDelta {C}_{tot}^{\left({R}_i-{R}_u\right)}={C}^{R_i}-{C}^{R_u}\\ {}=\varDelta {C}_{t, ES}^{\left({R}_i-{R}_u\right)}+\varDelta {C}_{t, EI}^{\left({R}_i-{R}_u\right)}+\varDelta {C}_{t, AS}^{\left({R}_i-{R}_u\right)}+\varDelta {C}_{t, AI}^{\left({R}_i-{R}_u\right)}+\varDelta {C}_{t, AG}^{\left({R}_i-{R}_u\right)}+\varDelta {C}_{t, TP}^{\left({R}_i-{R}_u\right)}\end{array}} $$

Based on the following equations, we can calculate the above effects on differences of CO_2_ emissions among cities in the transport sector.

11 $$ \varDelta {C}_{ES}^{\left({R}_i-{R}_u\right)}=\sum \limits_{j=1}^4\sum \limits_{k=1}^9L\left({C}_{ijk}^{R_i},{C}_{ujk}^{R_u}\right).\ln \left(\frac{F_{ijk}^{R_i}}{F_{ujk}^{R_u}}\right) $$

12 $$ \varDelta {C}_{EI}^{\left({R}_i-{R}_u\right)}=\sum \limits_{j=1}^4\sum \limits_{k=1}^9L\left({C}_{ij k}^{R_i},{C}_{uj k}^{R_u}\right).\ln \left(\frac{E{I}_{ij}^{R_i}}{E{I}_{uj}^{R_u}}\right) $$

13 $$ \varDelta {C}_{AS}^{\left({R}_i-{R}_u\right)}=\sum \limits_{j=1}^4\sum \limits_{k=1}^9L\left({C}_{ijk}^{R_i},{C}_{ujk}^{R_u}\right).\ln \left(\frac{A{S}_i^{R_i}}{A{S}_u^{R_u}}\right) $$

14 $$ \varDelta {C}_{AI}^{\left({R}_i-{R}_u\right)}=\sum \limits_{j=1}^4\sum \limits_{k=1}^9L\left({C}_{ijk}^{R_i},{C}_{ujk}^{R_u}\right).\ln \left(\frac{A{I}_i^{R_i}}{A{I}_u^{R_u}}\right) $$

15 $$ \varDelta {C}_{AG}^{\left({R}_i-{R}_u\right)}=\sum \limits_{j=1}^4\sum \limits_{k=1}^9L\left({C}_{ijk}^{R_i},{C}_{ujk}^{R_u}\right).\ln \left(\frac{A{G}_i^{R_i}}{A{G}_u^{R_u}}\right) $$

16 $$ \varDelta {C}_{TP}^{\left({R}_i-{R}_u\right)}=\sum \limits_{j=1}^4\sum \limits_{k=1}^9L\left({C}_{ijk}^{R_i},{C}_{ujk}^{R_u}\right).\ln \left(\frac{T{P}_i^{R_i}}{T{P}_u^{R_u}}\right) $$

where $L\left({C}_{ijk}^{R_i},{C}_{ujk}^{R_u}\right)=\frac{C_{ijk}^{R_i}-{C}_{ujk}^{R_u}}{\ln {C}_{ijk}^{R_i}-\ln {C}_{ujk}^{R_u}}$ is the logarithmic mean weight.

### Data source

In this study, we analyzed the CO_2_ emissions of China’s transport sector during 2000–2017 (due to the rapid development of the transport sector, the data before 2000 can be used for providing less reference in the current transportation research and development, and the data of energy consumption and CO_2_ emissions in the current accounts after 2017 are not available. So, we chose 2000–2017 as the study period) and divided it into four periods, i.e., 2000–2005, 2005–2010, and 2010–2017, which is catering to China’s economic development 5-year plans.

#### Energy consumption and CO_2_ emissions in China’s transport sector

The CO_2_ emission coefficients of different kinds of energy types came from the Intergovernmental Panel on Climate Change IPCC (2006), as shown in Table 4. The data of energy consumption and CO_2_ emissions for each city in China are collected from China Emission Accounts and Datasets (CEADs) (Shan et al. 2019).

**Table 4:** CO_2_ emission coefficients and fractions of carbon oxidized of different energy types

Fuel	F= CO_2_ emission	O=fractions of
	Factors, kg CO_2_/kg	Carbon oxidized, %
Coal	2.53	90
Coke	3.14	93
Crude oil	2.76	98
Fuel oil	2.98	98
Gasoline	2.20	98
Kerosene	2.56	98
Diesel oil	2.73	98
Natural gas	2.09	99
Electricity	0.90	–

#### Variables in socio-development

The data on annual GDP and population (the mean value of the beginning and end of the year) of each city during 2000–2017 have come from the statistical yearbook of the corresponding city.

The transportation services (passenger and freight) by transportation modes and energy consumption per transportation service are both collected from the statistical yearbook of the corresponding city. The transportation services are calculated with ton-kilometer in this study. For the convenience of statistics, the passenger person-kilometers must be converted to freight ton-kilometer through division by a conversion coefficient. The conversion coefficients for each transport mode refer to Wang et al. (2011), presented in Table 5.

**Table 5:** The conversion coefficient between passenger and freight ton (unit passenger/freight ton)

Transportation	Railway	Highway	Waterway	Civil aviation
Coefficient	1	5	3.03	13.88

## Results and discussion

### Analysis of CO_2_ emissions

**Fig. 2:**
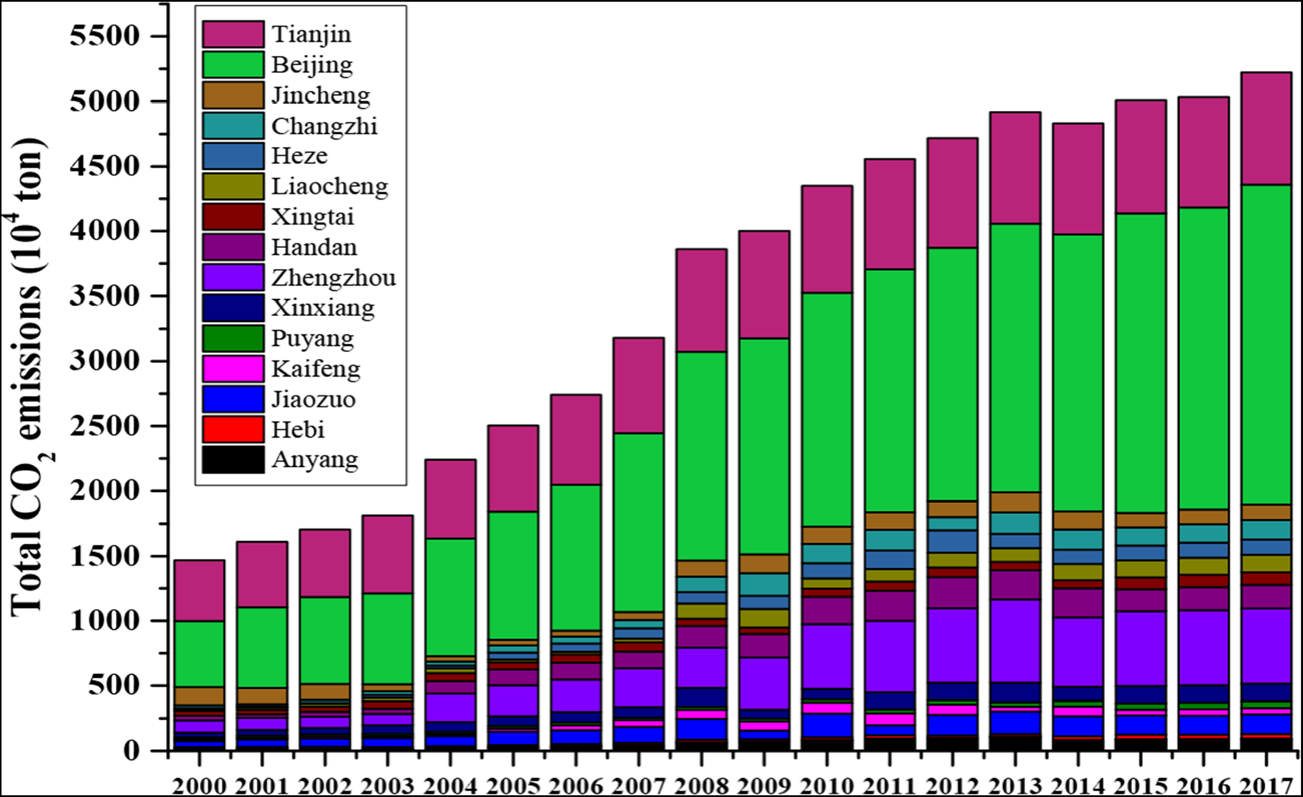
CO_2_ emissions of 15 east-central Chinese cities over time

Spanning 2000 to 2017, capital, municipality, and industrial cities had remained the vital contributors for most cumulative emissions (Fig. 2). But at the same time, CO_2_ emissions of 15 cities vary widely between the temporal changes. For instance, Zhengzhou (the capital of Henan), Beijing and Tianjin (the municipalities of China), Handan (an industrial city), and Jiaozuo (an industrial city) had produced the most cumulative emissions. However, Zhengzhou and Handan had experienced some fluctuations in total emissions. Beijing and Tianjin had been the largest contributors to CO_2_ emissions in all cities and the whole study period. Therefore, based on these results, there is a need to analyze the differences in CO_2_ emissions at the city level.

Moreover, some cities (i.e., Kaifeng, Anyang, Heze, and Xinxiang) experienced an emission peak in 2011 or 2013 according to the up-to-date results of this study. Therefore, exploring the historical trajectory and spatial differences of CO_2_ emissions in these typical cities could be crucial because this process could help cities grasp the trend and differences of CO_2_ emissions, so as to tackle climate change to reduce emissions (Tian et al. 2019).

**Fig. 3:**
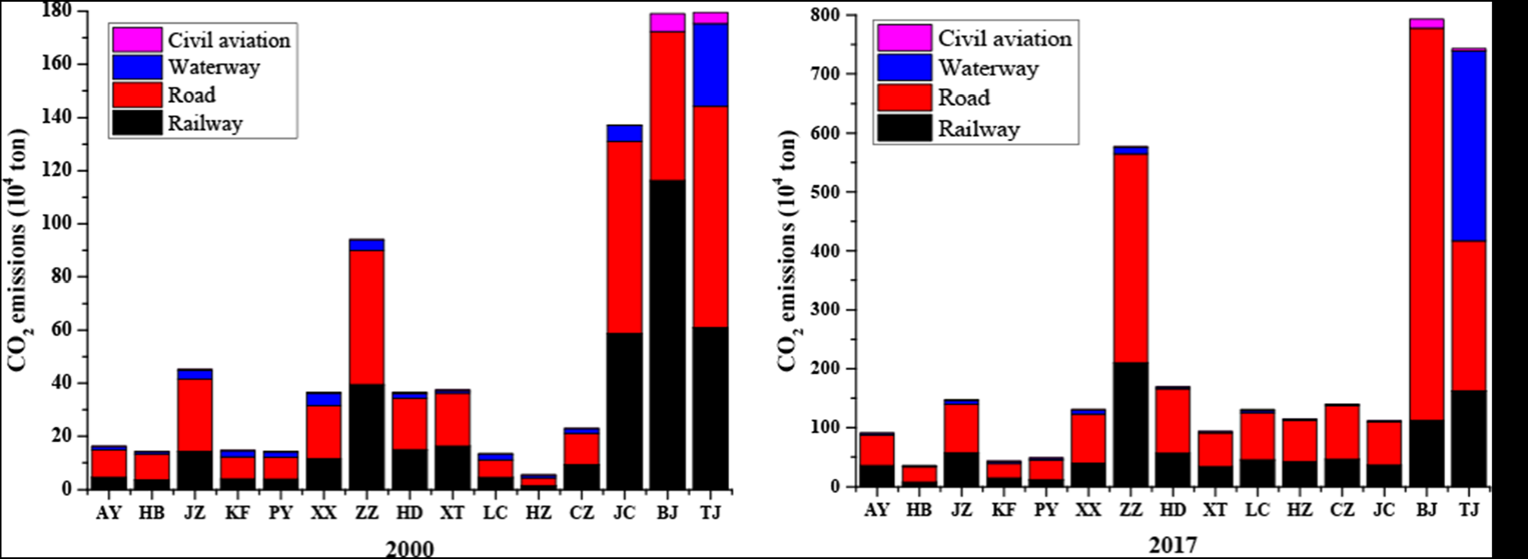
Share of CO_2_ emissions from different energy combustions in 2000 and 2017

A comparison of city-level CO_2_ emissions in 2000 and 2017 (Fig. 3) shows the detailed CO_2_ emissions from the transport sector in China’s 15 cities in 2000 and 2017, including CO_2_ emissions produced from sectors and energy types. From the whole point of view, the main source of CO_2_ emissions is generated from the road sector, followed by the railway; it is worth noting that the waterway sector emits more CO_2_ emissions than the road in Tianjin, and by contrast, there is no waterway in Beijing.

When it comes to the energy sources, we can know that the CO_2_ emissions from transport sector were induced from diesel and gasoline in most of cities and the share of diesel oil has showed a trend of growth from 2000 to 2017; i.e., the share of Handan, the city of Hebei province, is increased from 11.73 × 10^4^ tons to 131.15 × 10^4^ tons (approximately 68.23% of total emissions). However, the disparity of CO_2_ emission patterns exists from the city level. For instance, Beijing and Tianjin are important Chinese megacities, but with different CO_2_ emission patterns from the transport sector. In Beijing, the CO_2_ emission patterns from the transport sector were mostly contributed by kerosene (89.03 × 10^4^ tons), while in Tianjin, the consumption of both diesel and fuel oil contributed to most of the CO_2_ emissions from the transport sector (126.3 × 10^4^ tons and 101.7 × 10^4^ tons, respectively).

### Temporal decomposition analysis

**Fig. 4:**
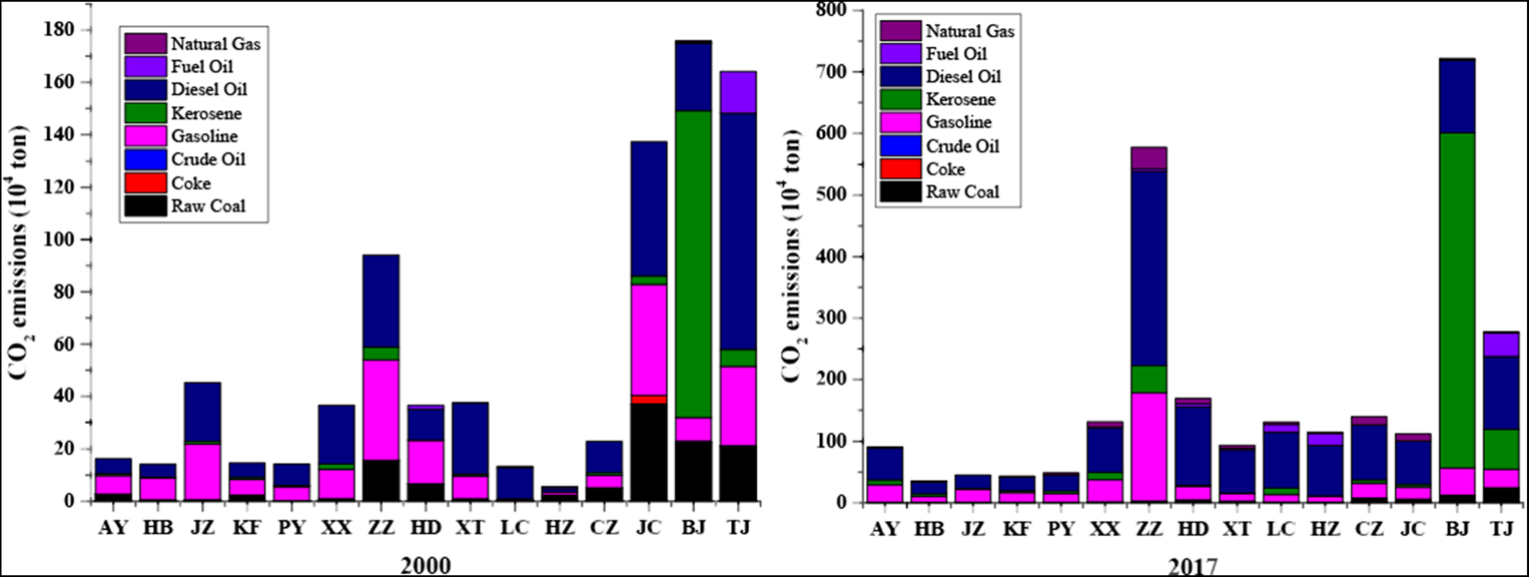
Share of CO_2_ emissions from different energy combustions in 2000 and 2017. *Note*: For the convenience of marketing, the name of the city is abbreviated, for example Anyang (AY)

For describing the hidden reasons for the changes of CO_2_ emission in the transport sector based on 15 cities in China, LMDI decomposition analysis was applied in the period of 2000–2017. Figure 4 promulgates total changes in CO_2_ emissions for the transport sector of 15 cities.

According to above, the contributions of various factors to CO_2_ emission are different during the study period; income effect (Δ*C*_*AG*_) was the dominant driving force that leads to the increase in city-level CO_2_ emissions while the transportation intensity effect (Δ*C*_*AI*_) was responsible for most cities to reduce the CO_2_ emissions, which were similar with previous literature, i.e., Wang et al. (2011); Achour and Belloumi (2016); Li et al. (2016); and Zhang et al. (2019). In addition, the population-scale effect (Δ*C*_*TP*_) also had a minor positive effect on CO_2_ emissions of the transport sector for the whole study period.

Δ*C*_*AG*_ played a significant role in increasing the CO_2_ emissions in most cities during each time interval; for example, Δ*C*_*AG*_ reached 869.23 × 10^4^ tons in Beijing during 2000–2017, next to Tianjin (668.14 × 10^4^ tons) and Zhengzhou (281.14 × 10^4^ tons), while the minimum contribution value reached 40.31 × 10^4^ tons in Anyang, next Jincheng (47.12 × 10^4^ tons), which is mainly because since the cities of Beijing and Tianjin are the municipalities and Zhengzhou is the capital for Henan province of China, the government paid more attention to promulgate policies for promoting economic activity development, which inevitably produces a lot of CO_2_ emissions (Zhang et al. 2019), while the cities of Anyang and Jincheng are less developed regions.

Δ*C*_*AI*_ was the key influencing factor that curbs the CO_2_ emissions in most cities during 2000–2017. With the rapid economic development in Henan province, the private cars also developed rapidly, in which its contribution value shows the tendency of fluctuating; Δ*C*_*AI*_ turned to be positive in specific cities of a special time interval, such as the time intervals of 2005–2010 and 2010–2017 in the cities of Anyang, Hebi, and Jiaozuo, respectively. The negative contribution value reached the largest − 102.03 × 10^4^ tons in Beijing; however, it was getting smaller during 2010–2017 (− 35.21 × 10^4^ tons), which mainly related to the developed high value-added industries (i.e., financial industries, service industries, research and development industries) in Beijing, where transportation output is higher than other regions (transportation services generated per unit of GDP).

Δ*C*_*EI*_ played an important role in increasing the CO_2_ emissions in some cities of Anyang, Puyang, and Changzhi during 2000–2015, which mainly attributed to the less-developed economy in the province of Henan and Shanxi, and energy use efficiency is lower than in developed provinces. In contrast, Δ*C*_*EI*_ had a positive influence on the time period of 2000–2005 of Beijing and Tianjin. This is in relation to Beijing and Tianjin having a more developed economy and high motorization rate than other regions, leading to traffic jams and high energy intensity.

Compared with other driving forces, the energy structure effect (Δ*C*_*ES*_) on CO_2_ emission changes from the transport sector is quite limited. Although, in some certain time intervals, Δ*C*_*ES*_ did augment CO_2_ emissions increasing in some cities, such as in Tianjin, Beijing, and Jincheng during 2010–2017, the contribution value is also minor, which may be due to the proportion of waterway which is getting larger (41.12%) in Tianjin, causing the demand of fuel oil (carbon-intensive energy) to increase. In contrast, the freight turnover of Beijing and Jincheng cities is increasing rapidly, and most of the freight turnover is mainly undertaken by large trucks and megatons; it consumes carbon-intensive energy (i.e., diesel) (Li et al. 2016; Zhang et al. 2019). According to Table 4, we can know that the fuel oil and diesel have higher carbon emission coefficient than other energy resources.

Δ*C*_*AS*_ was positive in most cities during 2000–2017, except for some special time period, such as 2010–2017. The contribution ofΔ*C*_*AS*_ reached the largest value in Tianjin (43 × 10^4^ tons), next to Handan (3.12 × 10^4^ tons) and Zhengzhou (2.08 × 10^4^ tons). The key reason was that the share of waterway in Tianjin is decreased from 39.36% in 2010 to 35.25% and with the rapid development and diversification of income groups, some comfortable and convenient transportation modes, such as road and civil aviation, provide more and more transportation services. In addition, the percentage of railways decreased substantially in Handan and Zhengzhou from 39.82% and 33.29% in 2010 to 34.62% and 30.27% in 2017, respectively. While the changes in Δ*C*_*AS*_ are helpful in reducing the total CO_2_ emissions of the transport sector during the same period in Beijing, they are mainly attributed to the optimization of transport structure, which leads to the proportion of high-speed railway and urban track construction increased (Zhang et al. 2019).

The effect of population scale (Δ*C*_*TP*_) had always been a positive factor in promoting CO_2_ emissions, but it played a relatively minor role. With Beijing reaching a maximum of 55 × 10^4^ tons, next Tianjin 44.52 × 10^4^ tons and Zhengzhou 32.47 × 10^4^ tons, this mainly attributed to Beijing, Tianjin, and Zhengzhou being more developed economy and have more job opportunities, which attracts a lot of external people and leads to an increase in transportation demand. In contrast, the contribution value of Anyang is the least, related to the backward economy and fewer motor vehicles (Li et al. 2017).

### Spatial decomposition analysis

In our study, the M-R spatial decomposition method was applied to investigate the factors causing the differences among 15 cities of China during 2000–2017. The average CO_2_ emissions at the national level are defined by arithmetic average CO_2_ emissions of total provinces of China, next to comparing the CO_2_ emissions of each city with the national level. Adopting the M-R spatial decomposition model to quantify the differences of the CO_2_ emissions and decomposing it into six influencing factors, the effect of each driving factor is calculated based on Eqs. (10)–(15). The positive and negative values of different factors have different meanings; for example, Δ*C*_*AI*_ is negative in some developed cities, such as Beijing and Tianjin, but it is positive in less-developed regions (Anyang, Puyang), indicating that Beijing had a higher transportation efficiency than the national average, while Anyang lower than the national average. Δ*C*_*AG*_ was positive in some developed cities (Beijing, Zhengzhou), which showed that the CO_2_ emissions due to economic output in developed cities are higher than the national average.

**Fig. 5:**
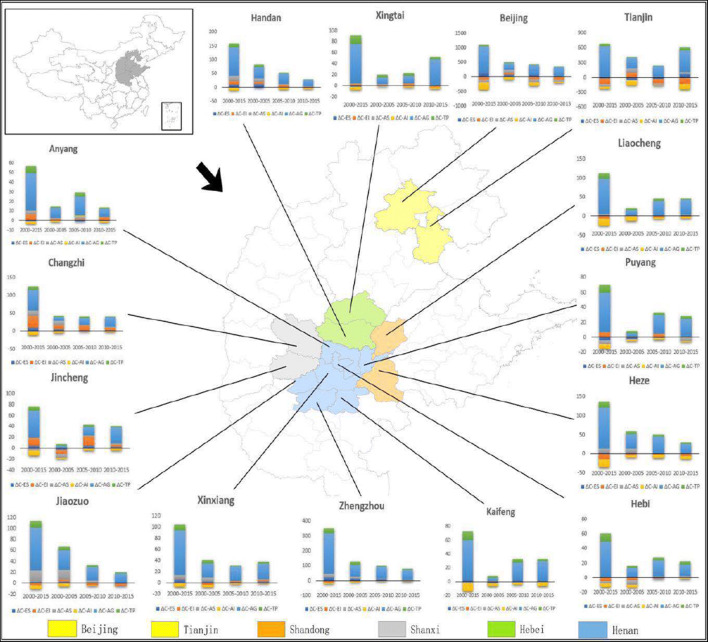
Temporal decomposition of the changes in CO_2_ emissions for each city at different time intervals

**Fig. 6:**
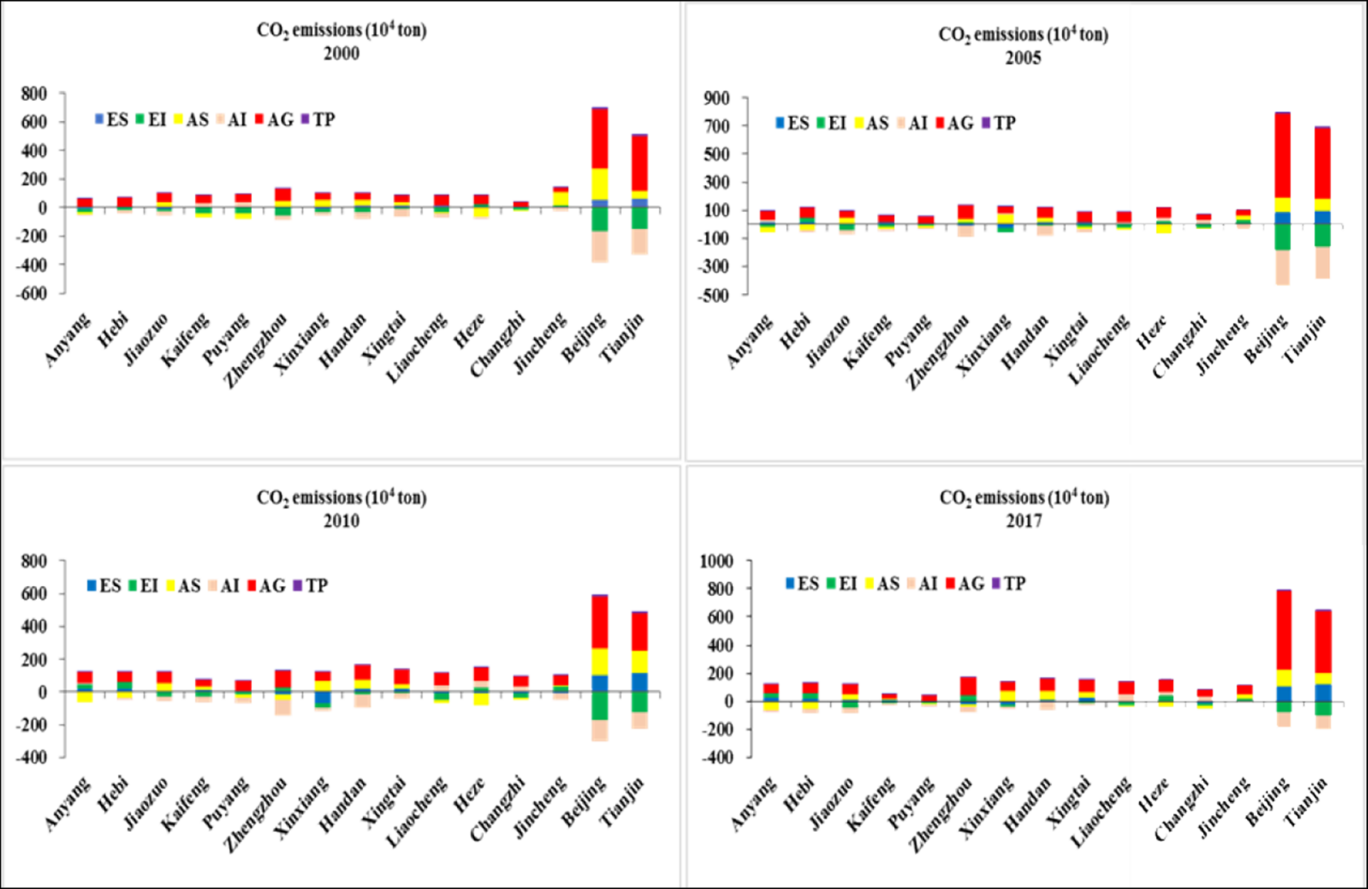
Spatial decomposition of city-level CO_2_ emissions of the transport sector in key years

Obviously, Δ*C*_*AI*_, Δ*C*_*EI*_, Δ*C*_*AS*_, and Δ*C*_*AG*_ were the most significant driving forces of differences in CO_2_ emissions in the transport sector between special city and the national average (Fig.5), while the other driving factors (Δ*C*_*TP*_and Δ*C*_*ES*_) played a minor role in causing the differences in CO_2_ emissions of 15 cities and the national average (Fig. 6).

Beijing, Tianjin, and Zhengzhou had the largest Δ*C*_*AG*_. The income effects of these three cities were highly greater than the national average level, and thus consumed larger energy resources and emitted amount of CO_2_ than the average. For example, Δ*C*_*AG*_ in Beijing reached 498.23 × 10^4^ tons in 2017, which was equivalent to higher 498.23 × 10^4^ tons than the national average. Yet, the income effect in cities like Anyang and Puyang was considerably smaller than the national average level, which resulted in the larger differences in economic scale among the cities.

Changzhi and Jincheng that belong to the Shanxi province, a highly resourced-based region, had a great higher Δ*C*_*EI*_ than the national average. Li et al. (2017) uncovered that Shanxi was a significant base of coal production and consumption and a less-developed technological level, which resulted in low energy efficiency and high energy consumption and CO_2_ emissions in the two cities. For example, Δ*C*_*EI*_ in Changzhi was 43.21 × 10^4^ tons, indicating that the high energy intensity in Changzhi leads to higher CO_2_ emissions of 43.21 × 10^4^ tons than the national level. In other words, Changzhi would decrease by 43.21 × 10^4^ tons if raising the energy using efficiency to the national average level. On the contrary, Δ*C*_*EI*_ in economically developed cities including Beijing, Tianjin, and Zhengzhou was far blew the national average level. It mainly attributed to the more developed technology level and higher energy efficiency.

Δ*C*_*AI*_ indicates the effect of the differences in transportation efficiency among cities, and it would decrease if the transport technology level is improved or the transportation goods with high value-added are developed. Highly developed cities(such as Beijing and Zhengzhou) that have more high value-added industries had a lower transportation intensity compared with the national average. In contrast, in highly undeveloped cities in which the agriculture sector is relatively large, the value-added is lower. For example, Δ*C*_*AI*_ in Hebi was 31.01 × 10^4^ tons in 2017, which revealed that high transportation intensity in Hebi increased CO_2_ emissions by 31.01 × 10^4^ tons than the national level.

Δ*C*_*AS*_ indicates the effect of the differences in transportation structure among cities, and it would decrease if the transportation structure were improved. Each transportation mode also varies significantly in energy structure and energy efficiency; the order is civil aviation > road > waterway > railway (Zhang et al. 2019). Beijing, Handan, and Zhengzhou had the largest and positive Δ*C*_*AS*_, which means that higher energy-intensive transportation modes, such as civil aviation and road, accounted for a relatively large proportion in these cities (Zhang et al. 2019), of which Beijing does not have the mode of waterway, the percentage of road occupied the more than half of the total, while the contribution value of Δ*C*_*AS*_was negative in some cities with a good waterway. For example, Δ*C*_*AS*_in Heze was − 30.14 × 10^4^ tons in 2017, which uncovered that perfect transportation structure (a large proportion of waterway) in Heze reduced CO_2_ emissions by 30.14 × 10^4^ tons than the national level.

However, the energy structure effect (Δ*C*_*ES*_) played a very minor role in the differences in CO_2_ emissions between the cities and the national average (Fig. 5). Zhengzhou had the largest negative Δ*C*_*ES*_ (− 18.28 × 10^4^ tons), indicating that Δ*C*_*ES*_ is lower than the national average level. It is mainly related to the fact that Zhengzhou is an important transportation hub, and the proportion of high-speed trains and railway, mainly consuming the clean energy of electricity, accounts for relatively large.

Δ*C*_*TP*_ is another less-influential factor in causing the differences in CO_2_ emissions between the cities and the national average. The Δ*C*_*TP*_ of some less-developed cities is lower than the national average, of which Anyang, Jiaozuo, and Xingtai had the largest negative contribution value of − 1.07 × 10^4^ tons, − 1.23 × 10^4^ tons, and − 1.89 × 10^4^ tons in 2010, respectively. This is related to the fact that these cities have less-developed economies and fewer job opportunities than the developed regions, resulting in a large number of people transporting from there to developed cities. Then, transportation demand in these cities decreases directly from population decrease (Zhang et al. 2019).

### Discussion

The main conclusion that emerged from our study is that the impact of income effect and transportation intensity on city-level CO_2_ emissions is quite large from the perspectives of temporal and spatial dimensions based on the LMDI and M-R models. We make a further discussion on one issue: What leads to similar emission trends at different emission scales? In a word, increasing energy consumption could attribute to production activities that it combusted to support the production of daily products and material (Achour and Belloumi 2016). In addition, economic development needs to the operation of equipment, which would result in energy consummation and inevitably lead to CO_2_ emissions. Thus, the emission scale is different; however, with the development of society, these cities are needed to shape their own economic scale, leading to similar emission trends at different emission scales.

### Policy implications

Our research results indicate that significant temporal and spatial disparities exist in CO_2_ emissions of the transport sector from the city level. The road sector has been leading the CO_2_ emission increase in the transport sector over the last decade, being responsible for about half of China’s total CO_2_ emission in the transport sector. Consequently, the road sector should be the key target in reducing CO_2_ emissions from the transport sector. It means that more comprehensive and stringent policies and standards should be firstly adopted in the road sector. First, the government should vigorously develop public transportation and urban trackless transportation, reducing the demand for a private car. Second, the government should improve power charging and natural gas equipment and facilities for encouraging citizens to buy electric cars and natural gas vehicles. Furthermore, economic instruments can be employed, such as increasing fuel taxes and charging emissions from vehicles (Guo et al. 2014).

There are differences in urban development; CO_2_ emissions from the transport sector in developed regions are larger than those in the less-developed regions, indicating the differentiated emission reductions for the city level of transport sector. Different cities should prepare their CO_2_ emission mitigation methods in the transport sector by considering the local realities, such as economically backward cities should learn from the developed regions to strengthen energy conservation and emission reduction through advancing energy-efficient technologies. The local government should provide financial support in scientific research and scientific research talents in less-developed cities (Guo et al. 2014; Zhang et al. 2019).

According to the LMDI and M-R decomposition results, the effects of transportation intensity and energy intensity particularly offset CO_2_ emission from the transport sector in developed cities, but contributed to CO_2_ emission increase from their transport sector in less-developed regions. Such results revealed that technology inequities and economic development models exist in the cities of China. So the backward cities should adopt the advanced energy-efficient technologies and vehicles to reduce energy intensity and improve economic development structure through giving priority to developing high value-added industries, such as the service industries and financial industries, to increase the value of transportation per unit of GDP.

## Conclusions

In this study, we investigate the CO_2_ emissions of the transport sector in China from the city level based on the temporal decomposition analysis model and spatial decomposition analysis model. Both the changes of CO_2_ emissions from China’s 15 cities and the differences of CO_2_ emissions between15 cities and the national average during 2000–2017 were investigated. The main conclusions drawn from this study are as follows:

1. Significant city-level disparities on CO_2_ emission features and driving factors exist in China’s transport sector from the temporal and spatial perspectives.
2. From the temporal perspective, income effect (Δ*C*_*AG*_) was the dominant driving force that leads to the increase in city-level CO_2_ emissions while transportation intensity effect (Δ*C*_*AI*_) was responsible for most cities in reducing CO_2_ emissions, and the population scale effect (Δ*C*_*TP*_) also had a minor positive effect on CO_2_ emissions of the transport sector for the whole study period. For example, Beijing reached the largest value of 869.23 × 10^4^ tons, while the minimum contribution value reached 41.03 × 10^4^ tons in Anyang in 2017; Δ*C*_*AI*_ shows a fluctuation turn during the study period, from positive on the time intervals of 2000–2005 to negative in 2005–2010 and 2010–2017 in the cities of Anyang, Hebi, and Jiaozuo, respectively. The negative contribution value reached the largest – 99 × 10^4^ tons in Beijing; however, it was getting smaller during the 2010–2017 (− 38.31 × 10^4^ tons).
3. From the spatial perspective, Δ*C*_*AI*_, Δ*C*_*EI*_, Δ*C*_*AS*_, and Δ*C*_*AG*_ were the most significant driving forces of the differences in CO_2_ emissions of the transport sector between special city and national average, of which the decomposition results can help us to understand what causes the differences in CO_2_ emissions of the transport sector among China’s 15 cities and then provide target mitigation methods for the city-level transport sector in the future.

## Data Availability

All the data and materials were freely available in the statistical Yearbook and CEDAs database.

## Footnotes

1 Two + twenty-six cities: namely Beijing, Tianjin, Shijiazhuang, Tangshan, Langfang, Baoding, Cangzhou, Hengshui, Xing tai, Handan, Taiyuan, Yangquan, Changzhi, Jincheng, Jinan, Zibo, Jining, Dezhou, Liaocheng, Binzhou, Heze, Zhengzhou, Kaifeng, Anyang, Hebi, Xinxiang, Jiaozuo, and Puyang
